# The Association between Sleep and Injury among School-Aged Children in Iran

**DOI:** 10.1155/2013/891090

**Published:** 2013-09-17

**Authors:** Forugh Rafii, Fatemeh Oskouie, Mahnaz Shoghi

**Affiliations:** ^1^Center for Nursing Care Research and School of Nursing and Midwifery, Iran University of Medical Sciences, Rashid Yasemi Street, Valiasr Avenue, P.O. Box 19395-4798, Tehran 19964, Iran; ^2^School of Nursing and Midwifery, Iran University of Medical Sciences, Rashid Yasemi Street, Valiasr Avenue, P.O. Box 19395-4798, Tehran 19964, Iran

## Abstract

*Background*. A good night's sleep plays a key role in diseases resistance, injury prevention, and mood stability. The objective of this study was to examine relationship between sleep problems and accidental injury occurrences in school-aged children. *Method*. A retrospective study was conducted for comparing two groups of children. Children who have experienced injuries for at least two times during an academic year are the participants in the injury group (IG) and those who have not experienced any kind of injuries are placed in the noninjury group (NIG). Data was collected through parent-reported sleep patterns and problems using Children's Sleep Habits Questionnaire (CSHQ). *Findings*. The findings showed that global sleep problems were more in the IG than in the NIG. Multivariate logistic regression analysis showed that the daytime sleepiness and sleep duration are the two major reasons for accidental injury. In addition, significant difference was seen between the sleep patterns of the two groups. Sleep duration was also shorter in the IG, and this group had a greater percentage (63% versus 41.1%) of “short sleepers” (<9 h). *Conclusion*. There is a significant relationship between injury occurrence and sleep problems and sleep duration in Iranian school-aged children.

## 1. Background 

Adequate sleep with regard to both quality and quantity is among the basic needs of human beings and plays a significant role in the physical, emotional, and cognitive development of children [1]. Sleep is regulated by complex and interacting biological and circadian processes, and sleep patterns also depend on physical, psychological, and environmental factors [2, 3]. Children's sleep patterns and habits in particular are influenced by external factors such as culture, health-related behaviors, parenting practices, and social interactions within the family. For example, caregivers' beliefs and their understanding of the meaning and importance of sleep may not only have direct effects on children's sleep patterns but may also impact parents' interpretation of sleep behaviors and their willingness to address problematic behaviors with their healthcare providers [2, 4, 5].

Sleep disturbances and irregular sleep patterns in childhood often lead to daytime sleepiness and short- and long-term consequences [2, 6], including somatic complaints such as headaches [7] and mood and neurobehavioral problems such as irritability, hyperactivity, and inattention [8–10]. The psychological and behavioral consequences may in turn result in increase in family and peer conflicts and parenting stress. Cognitive sequelae including poor impulse control and increased distractibility not only have potentially profound effects on academic achievement but may also have impacts on their life quality [11].

Many studies have been carried out regarding the relationship between sleep disturbance and injury in adults. for example, a number of studies have examined the relationship between insomnia and occupational [12–14] and driving accidents [15, 16] in adults. However, similar empirical evidence remains sparse for children [17–20]. In a study conducted in a pediatric emergency department, Valent et al. [20] reported a correlation between injury rates and sleep duration. In another emergency room study, Owens et al. [18] reported an relationship among accidental injuries, increased injury risk behaviors, and sleep problems in children [18]. Li et al. reached the same result in school-aged children [17]. Schwebel and Brezausek [19] study showed a relationship between the trauma occurrences and sleep disorders and night waking in toddlers [19]. 

Studies show that over the past twenty two years, deaths from accidents have been the second leading cause of death in Iranian children [21–23]. Iranian children were found to have more sleep problems compared to the American children and their sleep duration on average was 1.5 hour shorter than that of their American counterparts across all age groups. School start time is 8 a.m. in Iran and often children go to bed in irregular time at nights. Sleep time of children often is not very important for parents. Normally children and parents donot have different bed times and they usually sleep at the same time.

If sleep disturbance is associated with an increased risk of accidental injuries as the previous studies have shown, the increased rates of sleep problems in Iranian children may be one of the factors contributing to these high injury rates. Because little work has been conducted with respect to this potentially important and modifiable relationship between sleep behavior and injury in Iran, we conducted this study to explore the relationship between sleep problems and injuries in school-aged children from an urban area of Iran.

The aim of this retrospective case-control study was to examine the association between parent-reported sleep problems and accidental injuries in a sample of school-aged children in Iran. Our hypotheses were (1) children who have experienced accidental injuries at school will be more likely to have reported daytime sleepiness compared to children without injuries and (2) children who have experienced accidental injuries will have more parent-reported sleep problems compared to the noninjured group.

## 2. Methods

### 2.1. Subjects

All subjects were recruited from 17 elementary schools serving approximately 5000 students in Tehran, Iran. Participants were 400 school-aged children of 6–11 years old who live in a two-parent household in Tehran. Psychological disorders, chronic disease, ADHD, consumption of sedative or opioids/benzodiazepines/psychotropic drugs by children, and history of head trauma were considered as excluded criteria. In case of having any of the mentioned conditions in the child's health report in school or parents' reports during filling out the questionnaire, that specific child was not considered as a participant anymore. 

All Iranian children go through a medical test before they start any academic year and the report will be recorded and kept in the school. Children are insured at the beginning of the academic year. In case of any injury incidence in school, primary therapeutic actions will be performed by school nurses and in case of more serious injuries, the school authorities will inform the child parents and the child will be sent to the clinics under contract with the school for further treatments. Since the information just related to major injuries which is needed for referral is recorded in the children's health reports, minor injuries and harms that were not reported by the child have not been considered in this study.

The injury group includes those children who have reports about accidental injuries at school and who have faced accidental injuries for at least two times or more in the school during one educational year and have been sent to clinic for basic treatments by the school nurse. The noninjury group includes those children who are at the same age and they do not have any report concerning the injuries at school until the day of the administration of the questionnaires.

### 2.2. Instruments Procedure

 Two survey questionnaires were used in this study. The first questionnaire consists of two parts: a demographic section which includes variables such as child characteristics (age, gender,…) and parent characteristics (job, education,…). The second questionnaire deals with the questions about children's sleep problems and sleep patterns. The Children's Sleep Habits Questionnaire (CSHQ) is a validated parent-reported scale that assesses sleep behavior over a typical week and consists of 33 items grouped into eight subscales that reflect a variety of sleep domains: bedtime resistance, sleep-onset delay, sleeps duration, sleep anxiety, night awakenings, parasomnias, sleep-disordered breathing, and day-time sleepiness [24]. The CSHQ items are rated on a three-point scale: (1) “usually” if the sleep behavior occurred 5–7 times per week; (2) “sometimes” for 2–4 times per week; and (3) “rarely” for 0–10 time per week, with a higher score indicating more sleep problems. In addition, three questions regarding sleep patterns were asked about bedtime, morning wake-up time, and duration of daytime naps. Sleep duration was calculated by subtracting the reported bedtime and wake-up time, while nap duration was an actual time. Nine hours was used as a cutoff point for sleep duration in accordance to pediatric text books' recommendation of a minimum 9 h of sleep per night for this age group [25, 26]. 

This questionnaire was translated in to Persian previously and has been used in several studies concerning sleep habits in school-aged children in Iran. Content validity was used to estimate the scientific validity of this instrument. The reliability of the Persian version of this questionnaire was estimated as *r* = 0.87 using test-retest reliability coefficient [27–29].

*Procedure.* Receiving the letter of introduction from university and getting approval letter from Tehran Ministry of Education and Training, the researcher was introduced to public schools by this center. Having gone to the schools and having studied the children's health reports at the end of the educational year (June 2011), the researcher prepared a list of children who faced injuries for at least two times and were taken to clinics for further treatments (from September 23, 2010, to June 21, 2012). Those children who have experienced injuries which have happened within the last 15 days were considered as participants. After calling their mothers for a written permission, the researcher asked them to fill out the questionnaire in the school where both mothers and the child were present. The mothers were asked to fill out the questionnaire by focusing on the last time that their child had an injury and they also were asked to report the sleep patterns of their child one week before the occurrence of the injury. The injury group included 218 children. Ten mothers did not fill out the questionnaire and eight questionnaires were discarded due to incompleteness. All in all, 200 questionnaires have been left for analysis.

In order to avoid selection bias, the researcher chose stratified random sampling. The populations of boys or girls were chosen in the same age group and they were matched regarding their gender and age to compromise the noninjured (NIG). The questionnaires were administered and filled out in this group just like the previous group. The mothers were asked to fill out the questionnaire by focusing on the last time that their child had an injury and they also were asked to report the sleep patterns of their child one week before the occurrence of the injury.

### 2.3. Statistical Analysis

SPSS (version 15) was used for statistical analyses. The *t*-test analysis was used to determine the differences in sleep problem rates and daytime sleepiness between the two groups. Multivariate logistic regression analyses were conducted to examine the association between sleep problems (independent variable) and injury (dependent variable). Odds ratios (OR) and 95% confidence intervals (95% CI) were calculated. All statistical significance was set at *P* < 0.05.

## 3. Results 

200 children in the case group (62 girls, 138 boys) and 200 in the control group (78 girls, 122 boys) took part in this study. Gender distribution was similar across groups (*χ* ^2^ = 2.83, *P* = 0.93); the average ages of the control and case groups were 8.6 ± 1.73 and 8.4 ± 1.73, respectively, and not significantly different (*χ* ^2^ = 2.21, *P* = 0.89). Parental occupations were also similar. 81% of mothers were housewives in both groups and 35.7% of case group's fathers and 37.5% of control group's fathers were self-employed. Comparing two groups of case and control, using *χ* ^2^ test, the researcher did not find any difference regarding their age grade (*χ* ^2^ = 0.871, *P* = 1.83), mothers' education (*χ* ^2^ = 12.1, *P* = 1.17), fathers' education (*χ* ^2^ = 11.539, *P* = 0.061), mothers' jobs (*χ* ^2^ = 0.0, *P* = 1), and fathers' jobs (*χ* ^2^ = 12.11, *P* = 1.17).

### 3.1. Sleep/Wake-up Patterns

Mean bedtime at night for the total sample was 22 : 38 (standard deviation (SD): 1.57), mean morning wake-up time was 7.55 (SD: 1.07), and mean nocturnal sleep duration was 9.45 h (SD: 1.55). 

For the IG children, mean bedtime at night was 10 : 61 (SD: 1.98 h), mean morning wake-up time was 7.19 a.m. (SD: 1.13 h), and mean nocturnal sleep duration was 8.98 h (SD: 1.36 h). Sleep duration was calculated by subtracting the reported bedtime and wakeup time. For the control children, mean bedtime at night was 10.14 (SD: 0.94 min), mean morning wake-up time was 7.22 (SD: 0.88 min), and mean nocturnal sleep duration was 9.91 h (SD: 1.06 h). Of all three indices of sleep patterns, mean bed time at night (*t* = −5.68, *P* = 0.03), mean morning wakeup time (*t* = 6.56, *P* = 0.00), and nocturnal sleep duration (*t* = −6.23, *P* = 0.00) differed significantly between IG and NIG. Furthermore, 63.5% (*n* = 127) of children in the IG slept less than 9 h during the night, while only 41.1% (*n* = 87) of NIG children were reported to sleep less than 9 h (*χ* ^2^ = 20.29, *P* = 0.00). (*P < 0.05).

### 3.2. Comparisons of CSHQ

Regarding the second research hypothesis (children in IG would have a higher rate of sleep problems than children in NIG), the results were shown in Table 1.

### 3.3. Association of Injury with Sleep Problems in Injury Group

First, multivariate logistic regression analysis was conducted to examine the association between each subscale of the 8 individual sleep problems and injury. The results were shown in Table 2.

According to the findings of this study, these five categories of sleep problems have relationship with injury occurrence. The higher they go, the higher the probability of injury occurrence will be. Based on this model, the three categories of night waking, sleep duration, and daytime sleepiness have the greatest impact on injury occurrences (exep B).

## 4. Discussion 

Comparing the sleep patterns of the two groups, one can conclude that the children in IG sleep less than control group on average. They go to bed later than control group for various reasons and wake up earlier in the morning compared to control group. In addition, the number of short sleeper children (sleep < 9 hours) in IG was significantly greater than that of the children in NIG. Several studies have supported the mentioned results [17, 19, 20]; however Owens et al. [18], who did a research concerning children of 3–7 years old who had trauma, didn't find any significant relationship between the number of trauma occurrence and sleep duration [18].

The current study also suggests an association between sleep-onset delay, sleep duration, sleep anxiety, night wakenings, parasomnias, sleep-disordered breathing, day-time sleepiness, CSHQ total score, and accidental injuries. Li et al. [17] with similar results in their study showed that those kids with trauma experience in comparison with the ones without any experience suffer more from parasomnias, day-time sleepiness, sleep anxiety, and irregular sleep duration [17]. According to Owens et al. [18] sleep disturbance has significant relationship with the number of trauma occurrences and those children with more trauma experience suffer more from sleep disturbances such as bedtime resistance, sleep anxiety, and sleep-disordered breathing, compared to the children with less trauma experience [18]. This study supports other studies and shows that sleep anxiety is related more to trauma occurrence in school-aged children rather than other variables. Owens et al. [18] and Li et al. [17] reached similar results regarding this issue [17, 18].

The results of the current study support the existence of the relationship between sleep problems and injury occurrence in school-aged children. Perhaps exhaustion followed by parasomnias or the decrease of sleep quality was the reason concerning this issue [30]. Maybe decrease in the sleep quality and quantity could cause day-time sleepiness which would result in concentration loss, carelessness, and aggressiveness. Perhaps these conditions increased the risk of injury occurrence in school-aged children [6]. These findings do not appear to support cause-and-effect relationship between injuries and sleep problems.

It should be said that this study has some limitations. The mothers were asked to remember the sleep patterns of the week before the child's last injury while 15 days have passed since the last injury and this resulted in recall bias. Among the other factors that may have impacts on sleep pattern, one can refer to time of year. The concept of injury may vary among the parents and this should be considered as a factor as well. Since parents' definition may vary concerning the concept “injury,” their reports are very subjective and what one parent may consider as a minor injury may have been considered as major by another mother. Other factors such as parenting practices, family composition, and the environment should also be considered in future studies as influential variables. 

Sleep patterns may also vary in relationship to other factors, such as time of year. Variability in individual parent's thresholds for concern about injury may have also been a confounding factor in the analysis. Sleep information was based on subjective parental report. We lost data about minor and nonreported injuries. Finally, other factors such as family composition, household environment, and parenting practices may also play an important role in increased risk for injury and should be considered in future studies.

Despite its preliminary nature, the results of this study support an association between sleep disturbance and injury, among children in an urban area of Iran. Additional research is needed to further corroborate this relationship, using prospective designs, multiple observer reports of injuries and behavior, more detailed injury descriptions (e.g., time of day), and more objective and varied measures of daytime sleepiness. Our results underscore the need for screening and identification of sleep problems in children by healthcare providers and suggest that attention should be paid to sleep disturbance as a potential risk factor in children with increased injury rates. Both parents and school nurse should also be made aware of the possible association between sleep problems and injury risk as another potential consequence of inadequate and disturbed sleep.

## Figures and Tables

**Table 1:** Comparison of CSHQ scale scores between the IG and NIG means ± SD.

Sleep problems variables	IG (*n* = 200)	NIG (*n* = 200)	*t*	*P*
Bedtime resistance	11.88 ± 2.64	8.49 ± 2.08	14.23	0/00*
Sleep-onset delay	1.9 ± 0.78	1.32 ± 0.57	8.90	0/00*
Sleep duration	5.81 ± 1.48	4.11 ± 1.15	13.62	0/00*
Sleep anxiety	7.83 ± 1.97	5.38 ± 1.60	13.62	0/00*
Night waking	5.29 ± 1.44	3.69 ± 1.03	12.66	0/00*
Parasomnias	10.48 ± 2.33	8.09 ± 1.48	12.23	0/00*
Sleep-disordered breathing	4.46 ± 1.67	3.30 ± 0.8	8.79	0/00*
Daytime sleepiness	14.59 ± 3.26	10.45 ± 2.30	14.66	0/00*
Total scores	58.24 ± 9.84	41.99 ± 6.55	19.42	0/00*

*P < 0.05.

**Table 2:** Association of accidental injuries with sleep problem: multivariate logistic regression.

Sleep problem	*B*	SE	Wald	*P*	Exep (B)	95% C.I. for EXP (B)	
						Upper	Lower
Daytime sleepiness	−0.181	0.067	7.266	0.00	0.834	0.952	0.731
Bedtime resistance	−0.282	0.122	5.350	0.00	0.754	0.958	0.594
Parasomnias	−0.307	0.123	6.197	0.00	0.736	0.937	0.578
Night waking	−0.219	0.082	7.150	0.006	0.803	0.943	0.684
Sleep duration	−0.199	0.055	12.812	0.021	0.820	0.914	0.735

Daytime sleepiness entered on step 1; bedtime resistance entered on step 2; parasomnias entered on step 3; night waking entered on step 4; sleep duration entered on step 5.

*P < 0.05.
